# Pedestrian thermal comfort mapping for evidence-based urban planning; an interdisciplinary and user-friendly mobile approach for the case study of Dresden, Germany

**DOI:** 10.1007/s00484-024-02830-2

**Published:** 2025-01-03

**Authors:** Claire Gallacher, Denise Boehnke

**Affiliations:** 1 Leibniz Institute of Ecological Urban and Regional Development, Weberplatz 1, 01217 Dresden, Germany; 2 Faculty of Environmental Science, Dresden University of Technology, Dresden, Germany; 3 Institute of Geography and Geoecology, Urban and Mobility Geography, Karlsruhe Institute of Technology (KIT), Karlsruhe, Germany

**Keywords:** Thermal comfort, Urban planning, Meteorological device

## Abstract

In the face of climate change and increasing urbanisation, ensuring outdoor thermal comfort is becoming an increasingly crucial consideration for sustainable urban planning. However, informed decision-making is limited by the challenge of obtaining high-resolution thermal comfort data. This study introduces an interdisciplinary, low-resource, and user-friendly methodology for thermal comfort mapping, employing a self-built low-cost meteorological device for mobile climate monitoring. This device was utilised in the city center of Dresden, Germany to collect air temperature, humidity, pressure, surface temperature, global radiation, and globe temperature data as key inputs for the calculation of thermal comfort indices. These measurements were then used to calculate the Universal Thermal Climate Index (UTCI) using the RayMan Pro urban climate modelling program. Due to the limited resource capacities of urban planning departments, clear priority areas must be identified. Therefore, an exemplary approach for the prioritisation of consistent hotspots using the highest 5% of UTCI values was developed. The spatial variances in UTCI were validated through mobile pedestrian thermal comfort questionnaires, which allowed for the comparison of objective and subjective estimates of thermal comfort and gave the basis on which to make holistic and practical suggestions for urban planning interventions. This paper demonstrates an accessible and interdisciplinary approach to thermal comfort mapping which can empower urban planning stakeholders with scientifically informed and cost-effective decision-making tools for climate-adapted urban development.

## Introduction

Outdoor thermal comfort has emerged as a key consideration in sustainable urban planning. The urgency of this focus is amplified by the dual pressures of climate change and rapid urbanisation, which collectively pose profound challenges to the well-being of urban populations. While extreme heat may not frequently result in mortality within European contexts, thermal comfort is still inseparable from the broader discourse on urban liveability and quality of life (Lin et al. 2012; Nikolopoulou and Lykoudis 2007; Thorsson et al. 2004). Thermal comfort directly influences urban resident’s likelihood to engage in outdoor activities, social interactions, and participate in cultural events, thereby serving as a linchpin for economic vitality and communal health (Woolley 2003).

The role of local governments in addressing climate change and urbanisation through climate-adapted planning is becoming more prominent (Grafakos et al. 2019; Reckien et al. 2018). An important step in addressing local climate-related hazards is the development of climate action plans (Aboagye and Sharifi 2024). The success of creating such plans is highly dependent on the availability of data and tools that can provide evidence-based guidance (Aboagye and Sharifi 2024; Olazabal and Ruiz De Gopegui 2021). Therefore, addressing thermal comfort within the fabric of urban design presents challenges, notably due to the scarcity of high-resolution data that can accurately capture local-scale thermal variations in urban areas. As a result, due to data availability, decision-makers often rely on remotely sensed data to target interventions based on areas of high land surface temperature (Coutts et al. 2016).

The importance of using more holistic definitions of areas of heat stress for climate adaptation, going beyond mere land surface temperature, to include the use of thermal comfort indices which more accurately reflect human thermal experiences, is becoming more accepted within the context of urban planning. The understandability of thermal comfort indicators is key to their successful incorporation into urban planning, and to ensure this both the information and communication needs of decision-makers must be considered (de Groot-Reichwein et al. 2018). This underlines the importance of developing methodologies and visualisations that are both scientifically rigorous and easily interpretable to facilitate their use in creating practical insights for the implementation of evidence-based planning interventions and policy measures.

High-resolution thermal comfort data can be obtained through stationary urban micrometeorological networks or through carrying out micrometeorological measurement campaigns at the site of interest. Mobile meteorological devices may be mounted on vehicles (Kousis et al. 2021), carts (Middel and Krayenhoff 2019), or can be wearable (Chokhachian et al. 2018; Pigliautile and Pisello 2018; Ziemann et al. 2019) according to the specific goals of the investigation. In the context of urban planning assessments, mobile climate monitoring with pedestrian-carried devices offers the advantage of increased spatial resolution as well as access to pedestrianised areas which would not be possible with bulkier alternatives. However, data collection strategies that consider meteorological measurements alone fail to capture the nuances of human thermal comfort. Relatively few studies utilise a more holistic mobile meteorological approach that captures pedestrians’ natural walking routes through urban areas as well as their perceptions of transient conditions through questionnaires (Dzyuban et al. 2022; Lau 2022; Lemonsu et al. 2020; Peng et al. 2022; Vasilikou and Nikolopoulou 2020).

Within this context, this study demonstrates an interdisciplinary and low-resource approach to pedestrian thermal comfort mapping using exemplarily the case of Dresden, Germany. Mobile meteorological data were collected, using a built-for-purpose, low-cost, and user-friendly device, at four strategic times across six clear sky summer days from June-August, 2022. This data was used to calculate thermal indices using the RayMan climate modelling software. Mobile questionnaires were carried out in parallel to afternoon meteorological measurements to validate the use of the Universal Thermal Climate Indice (UTCI) in the context, as well as to identify and understand the differences in thermal hotspots and coldspots highlighted by subjective and objective assessments of thermal comfort.

Due to limited budgets and resource allocation, urban planning necessitates the prioritisation of specific areas for intervention (Boehnke et al. 2023; Fünfgeld et al. 2023). We therefore discuss how the results of this approach can be used to highlight priority areas for intervention and inform evidence-based urban planning measures. Exemplarily, the lowest 5% and highest 5% of UTCI values per lap of the monitoring route were used to spatially identify relative “hotspots” and “coldspots”, respectively, across the morning (06:00), midday (12:00), afternoon (15:30), and evening (20:00). To further prioritise areas for conservation and intervention, the coldspots and hotspots for each time of the day were intersected to find areas of consistent thermal comfort and discomfort.

The strength of this methodology lies in its accessibility and pragmatic focus, aiming to democratise the process of making thermal comfort assessments by empowering non-experts and urban planners with tools that require minimal prerequisite knowledge and resources. This is especially relevant in the context of new European climate adaptation regulations, particularly for the example of Germany after the adoption of the first nation-wide climate adaptation law in 2023 which requires risk analysis to be carried out, particularly at the local level, on which comprehensive adaptation concepts should be based (BMUV 2023). The approach presented in this paper, which focuses on smaller areas and urban districts where actions are already planned, aligns with the emerging needs of urban planners who seek actionable insights into how to prioritise areas for interventions and craft solutions that are both scientifically sound and contextually relevant. In doing so, this study bridges a critical gap in the literature, offering a framework that integrates quantitative meteorological measurements with qualitative insights from thermal comfort questionnaires to inform urban planning. This work ultimately aims to promote tools that are accessible and relevant for practical use and to present a way forward for the development of further innovative, low-resource approaches that can significantly enhance the vitality and liveability of urban spaces in the face of global climate challenges.

## Materials and methods

### Study site

The research was conducted in Dresden which is situated in the East of Germany, along the river Elbe. The city is classified under the Köppen-Geiger climate classification subtype of Marine West Coast Climate (Cfb), characterised by a temperate climate with an average temperature of 48 °F (8.9 °C) (Peel et al. 2007). Typically, July is the warmest month, with an average temperature of 65 °F (18.3 °C). However, during the year of the study, temperatures in July (2022) notably exceeded this average by 4.2 °F (2.39 °C), highlighting the significance of this study in the context of evolving climate patterns (Climate Data Center 2024b; Deutscher Wetterdienst 2022).

**Fig. 1:**
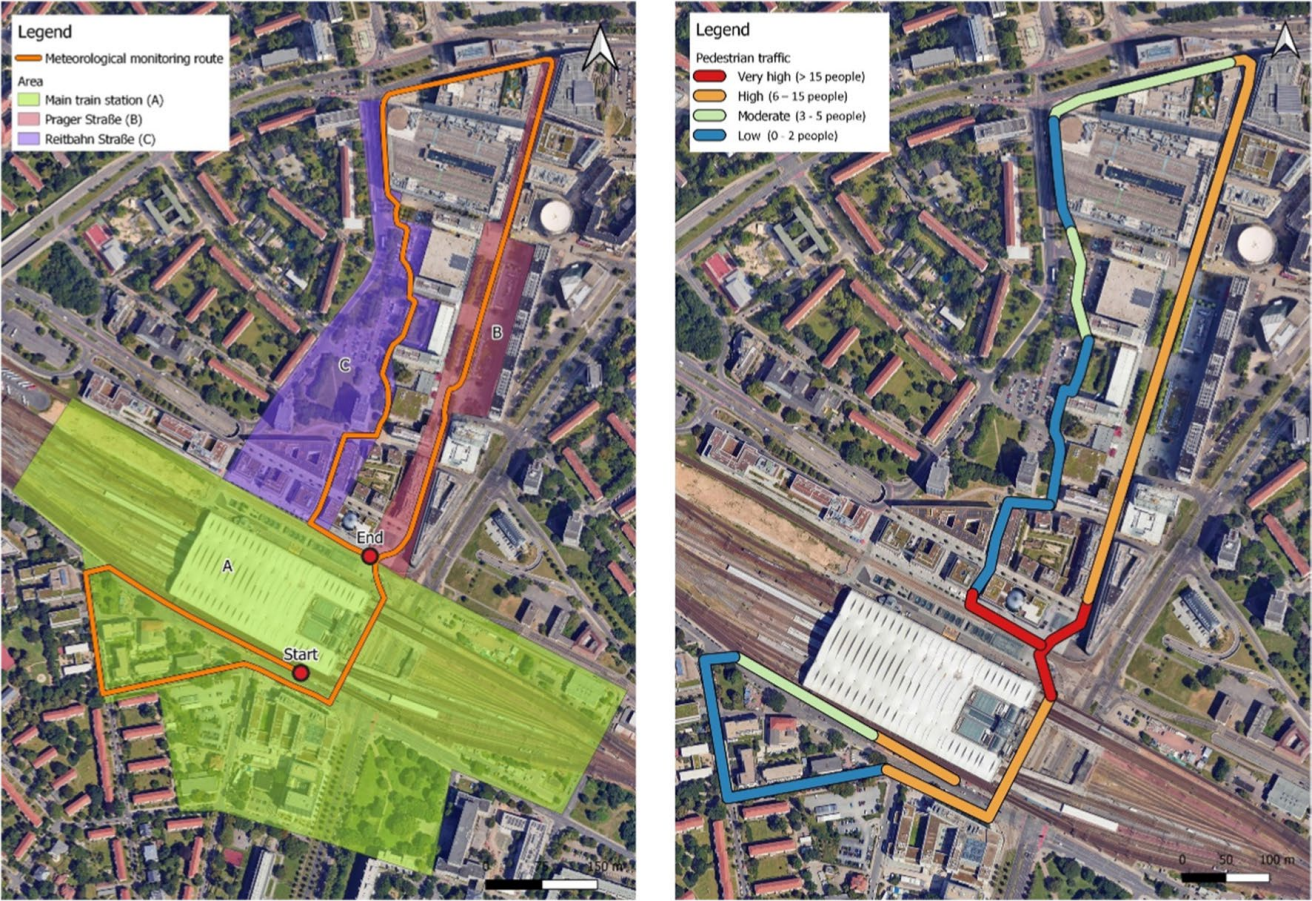
Context of the meteorological monitoring route (left) and subjective assessment of pedestrian presence (right) within the case study area of the city center of Dresden, Germany Base Map source © 2025 Google, Airbus, Maxar Technologies, GeoBasis-DE/BKG (©2009)

The study area was strategically selected within the city center of Dresden as it has been earmarked by the city’s Office of Urban Planning and Mobility for upcoming development projects. A survey was carried out by the city of Dresden within this area which identified that pedestrians were the most bothered by heat around the main train station and main shopping street (Landeshauptstadt Dresden 2023). Therefore, the study was designed so that the meteorological monitoring route traverses three distinct urban typologies; starting at the south entrance of the main train station (marked A in Fig. 1(left)), progressing north up the main shopping street named Prager Straße (marked B), and returning south through Reitbahnstraße which is another contrasting area with a parking lot, a small park, and residential buildings (marked C). The selection of this route aimed to comprehensively assess the thermal comfort conditions of the city centre within the areas of interest for the city’s Office of Urban Planning and Mobility, thereby offering diverse insights for urban planning.

A shorter segment of the meteorological monitoring route was identified for mobile thermal comfort questionnaires. The selection of this segment was based on the areas which are most frequently used by pedestrians. To this end, a qualitative assessment of local pedestrian presence was carried out in advance by walking through the route multiple times and noting the average number of pedestrians. For validation, this was also observed during the meteorological measurements from June to August (Fig. 1 (right)). As highlighted in Fig. 1, areas where an average of 2 or less people were visible are categorised as having “very low” pedestrian presence, an average of 3–5 as “moderate”, 6–15 as “high”, and greater than 15 as “very high”. All categories were approximated for the peak times of the day (during the midday (12:00) and afternoon (15:30) laps). All data was collected during June, July, and August of 2022, a period marked by heightened outdoor activities and the highest temperatures of the year in Dresden. The rationale behind this selection of both the study area and measurement period was to provide relevant data to guide evidence-based urban planning, thereby contributing to the creation of climate-adapted and liveable urban spaces for pedestrian activities.

### Meteorological measurements

To obtain precise meteorological data, crucial for assessing outdoor thermal comfort at the hyperlocal scale (10–30 m) relevant for urban planning (Venter et al. 2020), mobile meteorological measurements were systematically taken along an approximately 3.1 km long route, with each session lasting approximately 35 min, as depicted in Fig. 1 (left). Measurement campaigns took place on the 19th of June, 23rd of June, 19th of July, 25th of July, 16th of August, and 17th of August, 2022. The selection of these specific days were based on the presence of clear sky conditions which are essential for accurate global irradiation measurements as an important component of thermal comfort (VDI 2023). The afternoon laps on the 16th of August and the final evening lap on the 25th of July were therefore excluded due to unsuitable weather conditions. The predetermined route was traversed at eight times on each designated day, with measurements of two laps recorded starting at four critical time points: 06:00, 12:00, 15:30, and 20:00. This timing strategy was planned to capture a broad spectrum of thermal conditions throughout the day, thereby facilitating a comprehensive analysis of outdoor thermal comfort variability.

The measurements were carried out using a custom-built meteorological device, designed with reference to the German standards (VDI 2023) for resolution and accuracy while prioritising a low-resource and user-friendly approach by enhancing its portability and ease of use for single-researcher data collection. The device measures the essential variables for the calculation of thermal comfort indices, including air temperature, humidity, global irradiation, surface temperature, and globe temperature. Detailed information on the device’s components, as well as instructions on assembly, programming, and operation, are provided in (Gallacher 2024a  and b). The new device was successfully compared to two established, high-cost meteorological devices and a weather station in both stationary and mobile applications, demonstrating its ability to predict thermal comfort (Middel and Krayenhoff 2019; Ziemann et al. 2019; Moderow et al. 2023). Comparison data, available in (Gallacher 2024c, d; and e), confirm the suitability of the device for thermal comfort mapping.

The development cost was under 500 euros, with the pyranometer, as the most expensive component, accounting for approximately 200 euros of the total cost. The housing of the device and the main sensors are mounted on a GroPro stick, which can be carried in one hand. To begin data collection, the red button at the base of the device is pressed which flashes every 10 s to indicate that data is currently being collected. Once data collection is complete, the same red button is depressed and the microSD card can be removed from a slot in the side of the device and inserted into a computer to retrieve the data in Comma-Separated Values (CSV) format.

**Fig. 2:**
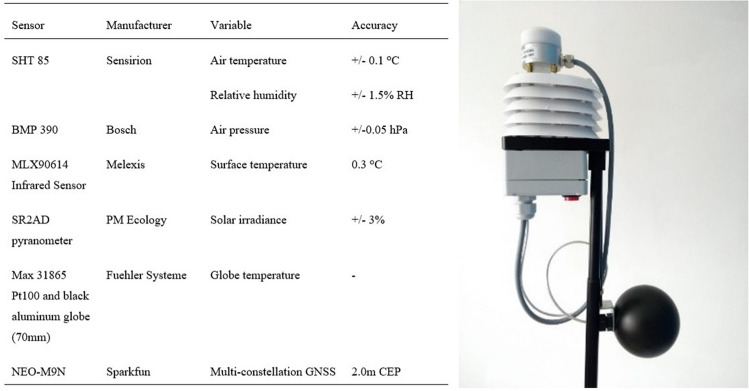
Device sensor components (left) and an image of the device (right)

Wind speed measurements were omitted due to the logistical challenges associated with mobile wind speed data collection and the negligible wind conditions prevailing in the study area during the measurement period. Equipped with Global Navigation Satellite System (GNSS) technology, the device ensures precise location tracking of measurement sites, with data recorded every second, using the GNSS to create a time stamp. Details of the sensors and a visual representation of the device are provided in Fig. 2, with more comprehensive information available in (Gallacher 2024a). The inclusion of visual data was facilitated through the use of a GoPro camera during the afternoon lap on July 19th which captured wide-angle fisheye images.

Analysis of the data gathered from each of the 45 laps of the mobile monitoring route was conducted using the RayMan Pro climate modelling program (Matzarakis et al. 2007, 2010). Default settings were used with the input location of Dresden at 13°41' E, 51°1' N with an altitude of 246 meters. The data collected with the device for calculating thermal indices were time, air temperature [°C], ground temperature [°C], relative humidity [%], and global irradiance [W/m²]. While data from the nearby DWD weather station “Dresden Strehlen” was considered for temporal calibration, it was deemed unnecessary due to the short duration of the monitoring route (approximately 35 minutes) and the specific goal of maintaining a user-friendly and resource-efficient methodology. Data from “Dresden Strehlen” showed air temperature variations of 0.8 K, 1.1 K, 0.6 K, and 1.2 K during the 35-minute walking route on June 19th at 6:00, 12:00, 15:30, and 20:00 respectively (Climate Data Center 2024a). However, for longer monitoring routes, temporal calibration may become more relevant to account for temperature fluctuations over extended periods.

The objective of this paper is to provide urban planners with straightforward and practical tools to identify relative thermal hotspots, enabling efficient allocation of resources for climate adaptation. Introducing additional complexity by incorporating temporal corrections would reduce the method’s practicality without significantly improving the results, especially given the intended application to urban planning.

While air temperature is an important factor for thermal comfort, it is one of several variables that contribute to indices such as the Universal Thermal Climate Index (UTCI). Given that other factors, such as global irradiance, play a more significant role in outdoor thermal comfort, minor corrections in air temperature from calibration would have minimal impact on the calculated UTCI values (Bröde et al. 2012; Fiala et al. 2012). To ensure sensor accuracy, the device was placed outdoors under shade for 15 min before each route, allowing for stabilisation prior to data collection.

### Questionnaires

Mobile pedestrian questionnaires were carried out which aimed to validate the selection of the thermal comfort index that would then be used in the rest of the analysis, as well as to capture differences between subjective thermal comfort assessments and objectively measured thermal indices within the study area. All questionnaires took place in the afternoon, starting at 15:30, to coincide with the period of highest thermal discomfort and pedestrian presence. The questionnaires were conducted in real-time alongside meteorological measurements to ensure direct comparison of subjective thermal comfort and objective thermal indices. The researcher carried the handheld meteorological monitoring device while accompanying the participants along the entire route.

**Fig. 3:**
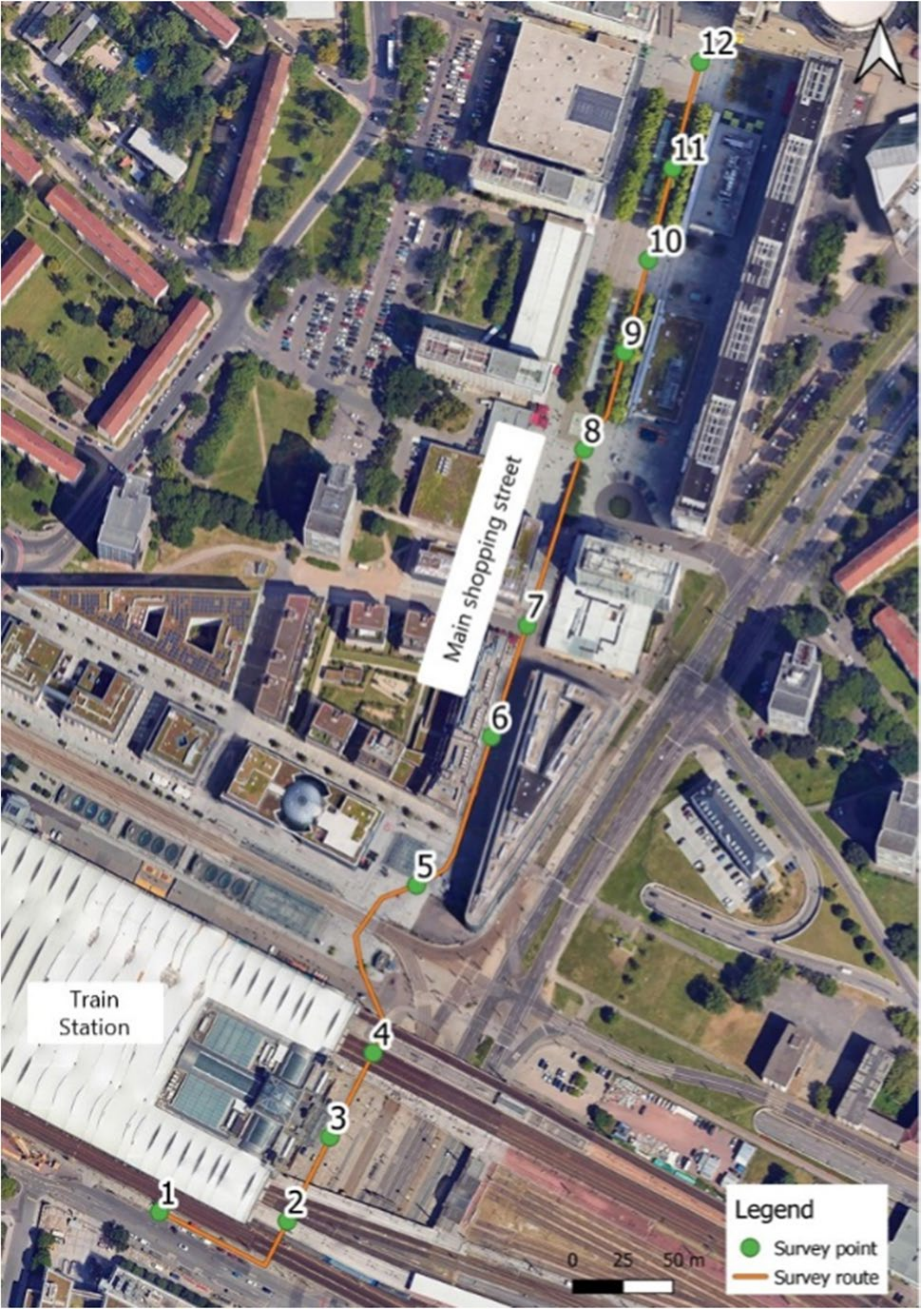
Survey points and route for thermal assessment using questionnaires Base Map source © 2025 Google, Airbus, Maxar Technologies, GeoBasis-DE/BKG (©2009)

A cohort of fifteen participants of mixed age (between the given ranges of 18 and 64 years old) and sex (7 male, 8 female) were recruited in advance to partake in this component of the study, with the criteria that they had lived in Dresden for at least two years prior. The questionnaire was first tested around the entire 3.1 km route with two participants, however, this was shortened after observing that after a certain point, the accumulated heat stress was too severe for participants to observe distinct changes in ambient stimuli. Thereafter, the route was shortened to a segment of the total meteorological monitoring route, approximately 800 m in length and 20 min in duration, which encompassed the vicinity of the main train station and the main shopping street (Fig. 3). The participants initially took around 60 s to answer the set of questions at the first stop, however this time decreased with each stop as participants became familiar with the questions and the procedure; translating to around 10 min walking time and 10 min for answering the questions overall. These areas were selected for their high pedestrian presence and highlighted thermal discomfort from the survey carried out by the city of Dresden (Landeshauptstadt Dresden 2023). Participants stood outside, under shade next to the starting point of the survey, for 15 min prior which allowed for adjustment to the outdoor conditions.

Participants were presented with a series of questions at twelve strategic points along the route which were selected for their diverse built environment characteristics (Fig. 3; Appendix see Table 2). The questionnaire was created according to the American Society of Heating, Refrigerating and Air-Conditioning Engineers Standard 55 (ASHRAE 2023) to incorporate the 7-point thermal sensation scale (ranging from hot (3) to cool (−3)), the 5-point thermal comfort scale (ranging from comfortable (2) to uncomfortable (−2)), and a question regarding thermal preference (if the participant would rather be (1) warmer, (0) no change, or (−1) cooler). The survey was designed to be accessible to all participants by offering both a paper format to be completed with a pen, and a digital format where participants could access the questionnaire, hosted by “SurveyMonkey”, via QR code or a web link using their personal mobile phones. The survey was made available in both the English and German languages.

### Data analysis

The questionnaire responses (*n* = 15) for thermal comfort and sensation were then compared statistically, using Excel functions, to thermal indices calculated by RayMan. For each of the 12 stops the average thermal comfort and thermal sensation vote was compared to the average of each of the thermal indices calculated by RayMan. Following this statistical analysis, the Universal Thermal Climate Index (UTCI) was found to be the most highly correlated index with both the thermal comfort (*p* = 0.72) and the thermal sensation votes (*p* = 0.81), as compared to other indices such as the Physiological Equivalent Temperature (*p* = 0.7, *p* = 0.79), and the Predicted Mean Vote (*p* = 0.66, *p* = 0.79). The UTCI was therefore identified as the most suitable thermal comfort index for assessing pedestrian thermal comfort in the context of Dresden’s city center and used for the rest of the analysis steps.

### Identification of hotspots and coldspots

For a comprehensive understanding of thermal comfort conditions, the analysis considered all meteorological measurement periods from morning (06:00) to evening (ending approx. 21:00). To identify the most thermally uncomfortable areas at each time of the day, the highest 5% of UTCI values were extracted for each lap of the meteorological measurement route. Thermal stress categories associated with UTCI should be first consulted for context (e.g., strong heat stress corresponding to 32 °C to 38 °C). However, the initial analysis found that these categories often highlighted very broad areas of high thermal stress, which did not provide meaningful insights into where targeted interventions should occur. The lack of granularity limited the ability to prioritise smaller, critical areas for intervention. Therefore, the 5% threshold was selected to target the most extreme cases of thermal discomfort while offering a manageable intervention area for urban planners. This threshold can be adapted based on the specific needs of the planning application, the size of the study area, available resources, and the capacity for intervention (Gallacher et al. 2024). While higher thresholds, such as 10%, were tested, they highlighted much broader areas, which are less practical for resource-constrained planners. In this case, the 5% threshold proved most suitable, as it highlighted more focused priority areas, enabling planners to allocate their resources more efficiently to the locations most in need of intervention.

These points were then visualised separately for all morning (06:00), midday (12:00), afternoon (15:30), and evening (20:00) laps. Points were snapped to align with the measurement route and then clustered into hotspots based on spatial proximity, while isolated points of three or fewer were omitted. The same method was used to identify relative coldspots, except the lowest 5% of UTCI values were considered at each time of the day. The final stage of geo-visualisation layered the clustered hotspots from the morning, midday, afternoon, and evening laps to highlight areas that were consistently thermally uncomfortable throughout all periods as consistent hotspots. The same process was again applied to the coldspot clusters to highlight consistent coldspots.

## Results

### General assessment of thermal comfort conditions in Dresden’s city center

The year 2022 was the sunniest and, together with 2018, the warmest year in Germany since systematic weather records began. The federal state of Saxony, where Dresden is located, recorded mean monthly temperatures of 18.9 to 19.8 °C in the study period from June to August 2022, which was 3.3/1.8/3.0 K hotter than the international reference period of 1961–1990. The total hours of sunshine were correspondingly high, ranging between 238 and 286 h/month from June to August, with a relative ratio to the reference period of between 120 and 142%. The precipitation deficit for the same period was 49.4%/55.6%/96% per month, compared to the reference period. For Saxony, official weather stations recorded 57.3 summer days (max > 25 °C) and 16.2 hot days (max > 30 °C), which were 183.4% and 295.7% compared to the reference period (1961–1990) (Deutscher Wetterdienst 2022).

The official weather station, “Dresden Strehlen”, from the German Weather Service, which is the station nearest to the study location, recorded 67 summer days (58 in June to August) and 24 hot days in 2022, which are the highest numbers on record, apart from the exceptionally hot year 2018 which had 94 summer days (63 from June to August) and 39 hot days (32 from June to August). Daily temperature means for 2022 were 20.6 °C in June, 20.7 °C in July, and 21.2 °C in August, and maximum daily temperature values averaged 26.9 °C in June and July and 27.4 °C in August (Climate Data Center 2024b) .

The general assessment of the heat load using the UTCI, based on the local meteorological measurements on 6 clear-sky summer days across June, July, and August 2022, is correspondingly high as illustrated in Table 1. Due to the night-time cooling of air temperature and weaker solar radiation, the heat stress is lowest in the morning (moderate heat stress of 26 to 32 °C). Weaker solar radiation with higher air temperatures leads to the categorisation of strong heat stress in the evening (32 to 38 °C). Strong to very strong heat stress occurred during midday measurements, where there was the highest global irradiance, with the maximum heat stress observed during the afternoon laps of the measurement route with the highest accumulated air temperature. The meteorological data collected as part of this study as well as the thermal indices calculated with this data using RayMan are fully available at (Gallacher 2024f).

**Table 1:**
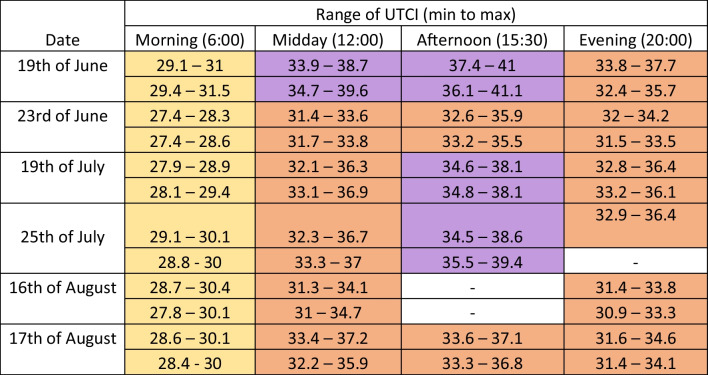
Range of UTCI measured on all 45 laps of the measurement route. UTCI from 26 to 32 °C (yellow) indicates moderate heat stress, 32 to 38 °C (orange) strong heat stress and 38 to 46 °C (purple) very strong heat stress (Blazejczyk et al. 2012)

### Comparison of UTCI to the thermal comfort and thermal sensation vote from questionnaires

**Fig. 4:**
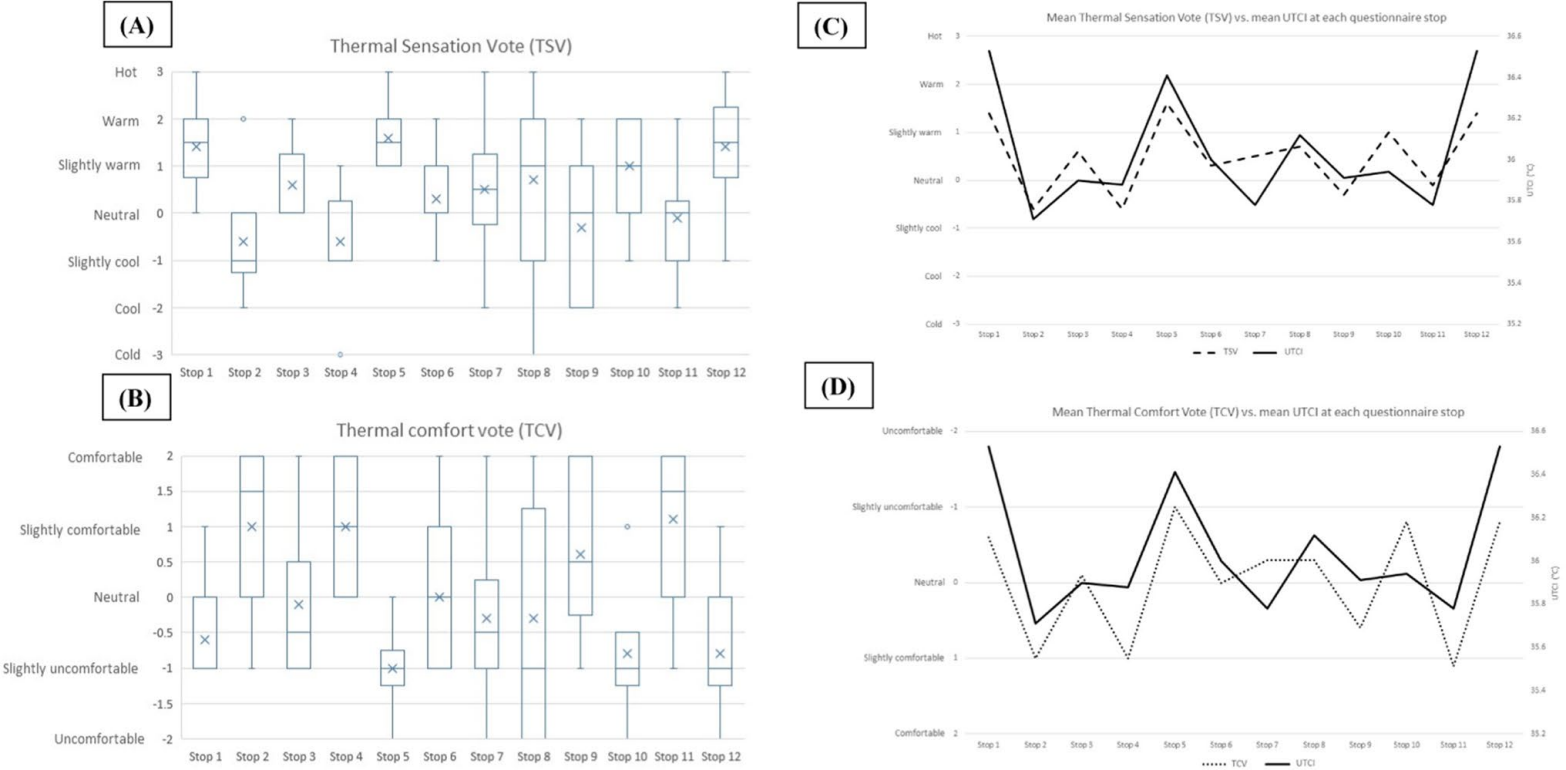
Analysis of (**A**) Variance in Thermal Sensation Votes at each stop, (**B**) Variance of Thermal Comfort Votes at each stop, (**C**) Comparison of mean Thermal Sensation Vote vs. mean afternoon (15:30) UTCI at each stop, and (**D**) Comparison of mean Thermal Comfort Vote vs. mean afternoon (15:30) UTCI at each stop

As highlighted in the previous section, UTCI values were highest during the late afternoon measurements (15:30 local time), identifying this as the period of highest heat stress for pedestrians. The questionnaires were conducted exclusively during the afternoon sessions, in parallel to the meteorological measurements for comparison. Figure 4 illustrates the variability between questionnaire participants at each of the 12 stops for the thermal sensation vote (A) and thermal comfort vote (B). The responses of all interviewees (15 total) across all survey dates were combined into one boxplot for each stop (1 to 12). For thermal sensation, a value of 3 represents the highest temperature, corresponding to the descriptor “hot”. For thermal comfort, a value of −2 represents the descriptor “very uncomfortable”. Stop 8, positioned next to a water feature, showed the greatest variation in both thermal comfort and thermal sensation votes, while Stop 5, located in an open, sun-exposed square, showed the least variation.

Thermal comfort votes exhibited greater variance at each stop compared to thermal sensation, reflecting the broader psychological and subjective nature of comfort. While both thermal comfort and thermal sensation are subjective and depend on individual perceptions, thermal comfort involves more complex factors such as psychological expectations and personal preferences. This distinction was first introduced by Fanger (1970), who emphasised that thermal comfort is a state of mind reflecting satisfaction with the thermal environment, beyond the immediate perception of warmth or coolness. These findings have been further supported by more recent studies (Wagner 2007, Karjalainen 2012; Humphreys and Nicol 2002).

The questionnaire results were also compared to the UTCI for validation of the spatial identification of hotspots and coldspots, as well as for the identification of points of discrepancy between objective and subjective assessments of thermal comfort, indicating where a more detailed assessment should be carried out. The mean UTCI value at each stop was compared to the mean thermal sensation vote (Fig. 4C), and the mean thermal comfort vote (Fig. 4D), graphically and statistically. The correlation analysis supports the visual assessment, presenting a high correlation between UTCI and both the thermal sensation vote (*r* = 0.81) and the thermal comfort vote (*r* = 0.72). The higher statistical correlation with the thermal sensation vote can again be explained by the more individually subjective nature of thermal comfort. Only at Stop 7 and 10, both the thermal comfort and thermal sensation votes were relatively higher than the UTCI. These two stops were both in sun-exposed areas immediately following a survey stop located in a shaded urban canyon (Stop 6) and a pedestrian walkway shaded by trees (Stop 9). Based upon correlative analysis for each stop it was derived that the pedestrian participants had a neutral thermal comfort vote (0) within the UTCI range of 35.7–36.5 °C.

### Identification of target areas; consistent hotspots and consistent coldspots

**Fig. 5:**
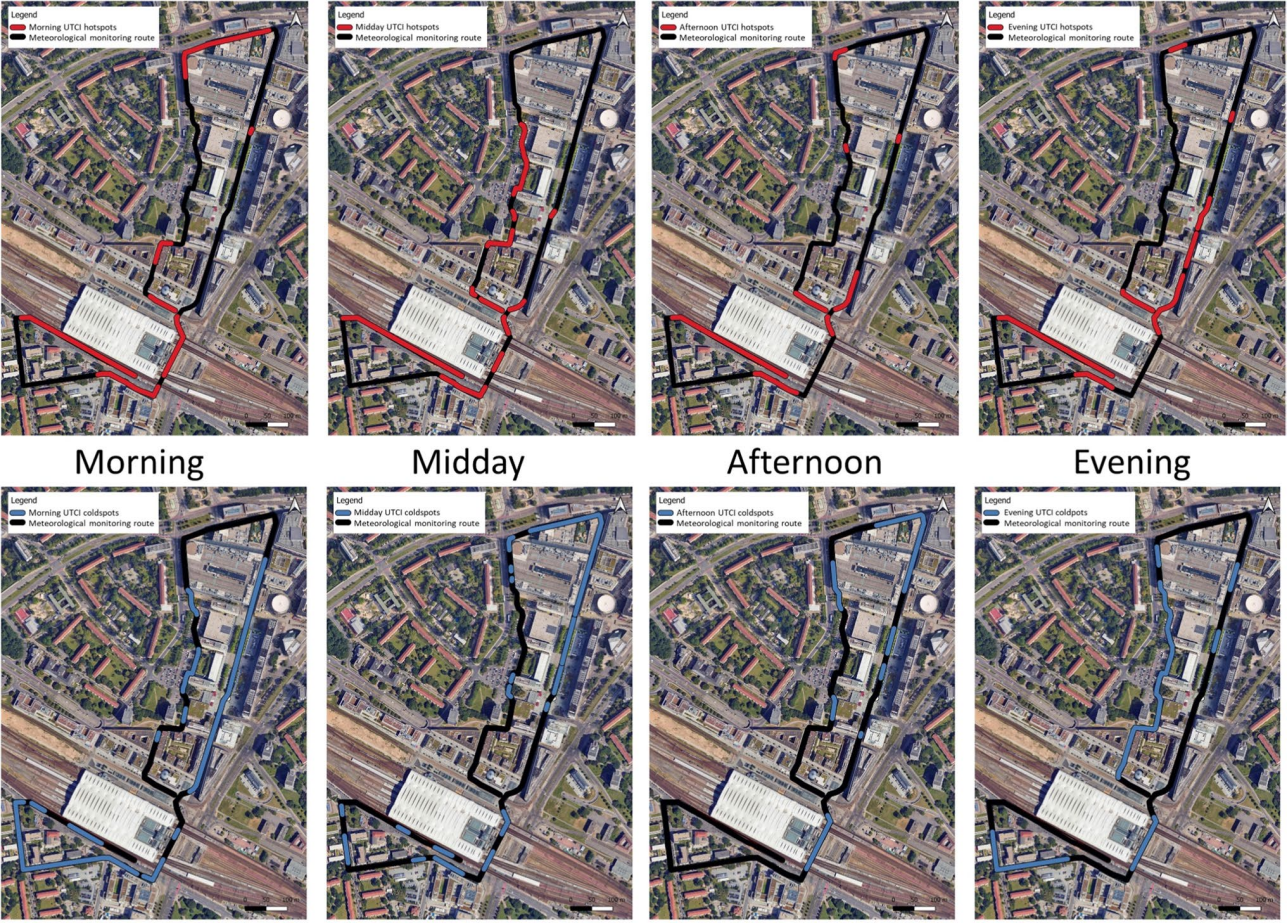
Clustered hotspots and coldspots visualized along the meteorological measurement route Base Map source © 2025 Google, Airbus, Maxar Technologies, GeoBasis-DE/BKG (©2009)

The results presented in Fig. 5 show the clustered hotspots and coldspots post-processing, after they had been snapped to the mobile monitoring route for better visualisation accuracy. Both the hotspot and coldspot locations are shown to move throughout the day, primarily due to variations in global irradiance. Particularly in the urban canyon of the main shopping street, hotspots and coldspots were shown to vary throughout the day according to sun exposure.

**Fig. 6:**
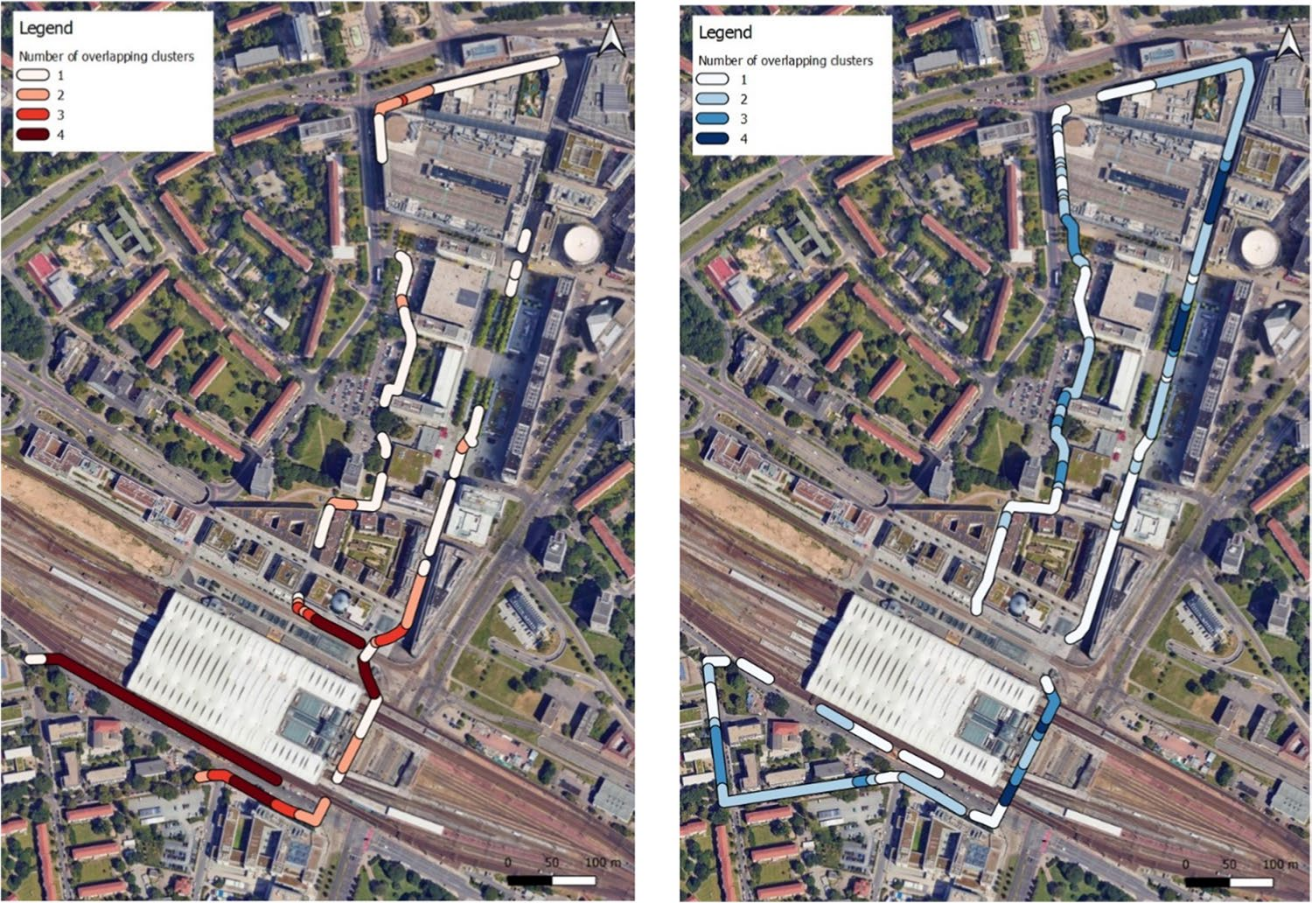
Consistent hotspots and coldspots Base Map source © 2025 Google, Airbus, Maxar Technologies, GeoBasis-DE/BKG (©2009)

Figure 6 (left) shows the locations of four consistent hotspots, highlighted in dark red, where there was the presence of the top 5% of UTCI values at all four times of the day. Figure 6 (right) shows four consistent coldspot locations, highlighted in dark blue, in which there was the presence of the lowest 5% of UTCI values at all four measured times of the day.

**Fig. 7:**
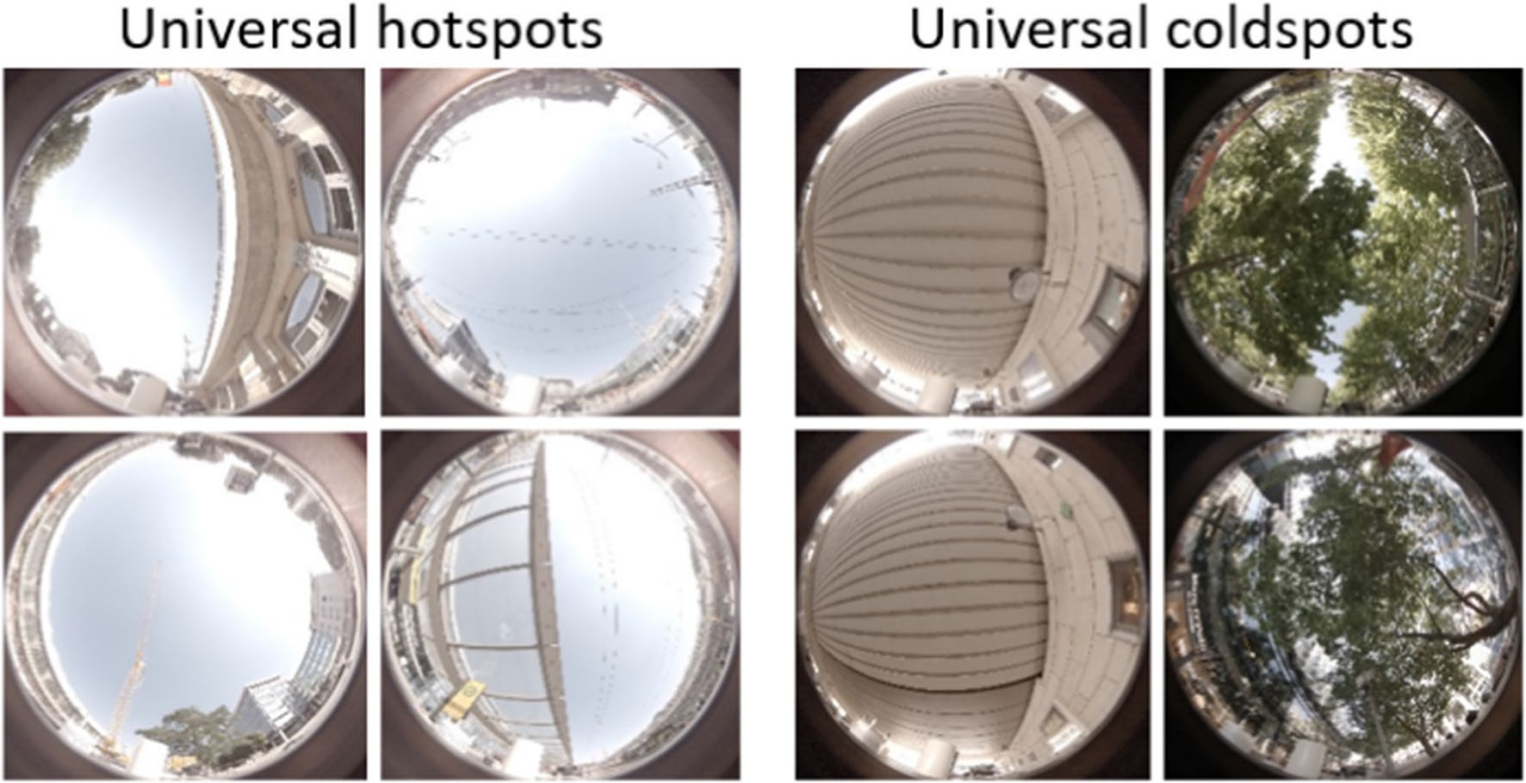
Fish eye images of consistent hotspots and consistent coldspots (Fish eye image credit: Dr. Valeri Goldberg, TU Dresden)

The four areas with consistent hotspots are identified as priority areas for climate-adapted interventions. These areas are mainly characterised as areas with little to no shading throughout the day, with 3 out of 4 located at the southern façade of buildings (Fig. 7; Appendix see Table 3). In contrast, the areas with consistent coldspots are characterised by pronounced tree shadow and/or pronounced building shadow, with two coldspot areas located below a railway bridge (Fig. 7; Appendix see Table 4).

Areas which were identified as consistent UTCI hotspots, closely aligned with the findings of the subjective assessment of thermal comfort from the mobile questionnaires. The thermal sensation vote and the thermal comfort vote indicated thermal hotspots at stops 1, 5, 10 and 12. Stops 1 and 5 were similarly indicated as consistent UTCI hotspots in Fig. 6. Stop 12 was also identified within the top 5% of UTCI values in the morning (06:00) and afternoon (15:30) laps. However, stop 10 was not identified as a hotspot during any period when considering UTCI for the entire mobile monitoring route. Stops 2, 4, and 11 which were identified as the most thermally comfortable points of the questionnaire route were all identified in Fig. 6 as consistent coldspots. The fourth consistent coldspot, identified in Fig. 6, was not included within the scope of the mobile questionnaire route.

## Discussion

### Prioritisation of target areas

With the application of thermal comfort mapping in informing action plans for decision-makers, it is important to communicate priority areas for action due to limited resources (Fünfgeld et al. 2023; Rogers et al. 2023). The approach outlined in this paper provides a template for decision-makers to prioritise areas for action where they can argue for the implementation of heat measures. In this case the UTCI with a 5% threshold for prioritisation was applied; however, depending on the needs of the decision-makers, this threshold should be altered (Gallacher et al. 2024). Another method could consider ranking the urgency of intervention based on the stipulation of multiple UTCI thresholds of 5%, 10% and 20%, for example. For the definition of the applied approach within a specific planning context, it is crucial to consider both the communication needs and information needs of decision-makers in an iteratively developed methodology (de Groot-Reichwein et al. 2018; Gallacher et al. 2024).

In this approach, consistent hotspots were highlighted for intervention which are areas indicated as hotspots across all measurement times: morning (06:00), midday (12:00), afternoon (15:30), and evening (20:00). However, the times of midday and afternoon were shown to have the highest thermal comfort index category, with some periods of very strong heat stress, and therefore the hotspots highlighted at these times should be further prioritised when considering pedestrian exposure. In consideration of the maintenance of pedestrian thermal comfort, coldspots which are especially identified during times of the most critical heat stress, i.e. during periods of highest solar radiation (12:00) and air temperature (15:30) are of special interest and conversely should be prioritised for conservation.

Within the framework of risk assessment, the heat hazard itself (represented in this case by the UTCI) is a single component of risk, and exposure and vulnerability should also be considered in the identification of target areas for interventions (Dong et al. 2020; Maragno et al. 2020; Scherer et al. 2013). The approach of prioritising the consistent hotspots, ensures that the duration of exposure to thermally uncomfortable conditions throughout the day also considered. Areas with high foot-traffic (e.g. the main shopping street) and long idle durations of pedestrians (e.g. public transport stops) should be further prioritised as critical points for intervention in order to have the greatest impact on public health and wellbeing. Furthermore, an assessment of land use should also be incorporated in the definition of areas for intervention, which would facilitate a targeted prioritisation which also considers the feasibility of implementing specific interventions based on ownership.

The differences between the hotspots and coldspots as highlighted by the UTCI and by the thermal comfort and thermal sensation votes from the questionnaires should also be considered in prioritisation. The objective and subjective assessments of thermal comfort was found to be largely in agreement. However, areas of discrepancy should be analysed in more detail to ensure appropriate and user-accepted interventions.

### Relevance for evidence-based decision making

From a planning and administrative perspective, further aspects need to be considered when selecting specific areas for action and feasible measures for intervention. An identified hotspot may not require any measures if alternative shading options (trees, public buildings, technical shading) or even publicly accessible air-conditioned rooms are available within a short walking distance of the hotspot location. In principle, greening measures are preferable to technical measures, as in addition to thermal cooling they also provide various ecosystem services (Boehnke et al. 2022) and trees provide the most benefits regarding their local cooling potential (Wong et al. 2021). In multiply-used inner city areas, however, it is only possible to unseal areas and plant trees to a very limited extent.

The results clearly show that thermal comfort depends crucially on shading and incoming solar radiation. The consistent hotspots are characterised by full exposure to solar radiation, while the consistent coldspots are located in solidly roofed pedestrian tunnels and under trees with dense vegetation. These findings are in line with many studies which provide evidence for the positive effect of a strong reduction of solar radiation due to dense tree canopies (e.g. Huang et al. 2020; Lee et al. 2018; Wong et al. 2021).

When selecting measures, interventions that reduce radiation exposure should therefore be prioritised. Urban tree species with dense foliage (i.e. high normalised difference vegetation index (NDVI)) that are suitable despite climate changes should be selected to provide direct shade for pedestrians. Dense, healthy façade greening significantly reduces the reflection of solar radiation from the façade into the street space by utilising a large proportion of the incident radiation for photosynthesis (Wong et al. 2021). From a human biometeorological perspective, they would be favoured measures along highly frequented routes such as the main shopping street; however, façade greening is only easy to maintain under certain technical conditions (e.g. few windows). The installation of green façades in existing buildings is also difficult to achieve under planning law, and in principle even more so in public buildings than in private buildings (Boehnke et al. 2023).

The approach presented serves to support decision-making for the selection of specific locations with a particular need for action (hotspots) or which are particularly worthy of protection (coldspots). The user-friendly approach to data collection advocated for in this study aims to democratise the process, making it feasible for non-experts to contribute to the gathering and analysis of relevant data. Accordingly, this approach is particularly valuable for resource-constrained smaller to mid-sized municipalities (Fünfgeld et al. 2023), in addition to larger cities with more resources and personnel such as Dresden.

The focus of the method lies in smaller areas and urban districts for which actions are already planned and which require specific data for alternative analyses, which are easily accessible and low-cost. For large-scale urban climate studies other methods are preferable, e.g. simulations, modelling, or remote sensing analyses (Kim and Brown 2021). Modelling and simulations can also be used at the microscale, however, they require far more input data, and a high level of software expertise, and place high demands on interpretation for the selection of specific focal points for action (Masson et al. 2020).

The strength of the approach presented lies within the ability to provide location-specific assessments of summer heat-stress and thus provides a concrete argumentation basis for urban planners - an important prerequisite for justifying climate-relevant needs in the planning process and advocating for them against other interests (Boehnke et al. 2023; Rogers et al. 2023). By identifying specific areas of thermal discomfort and understanding the contributing factors, planners are equipped with concrete data with which to inform evidence-based interventions. This evidence-based methodology not only aids in the prioritisation of the intervention areas but also ensures that the chosen solutions are directly responsive to the identified needs, thereby enhancing the efficacy and efficiency of urban planning efforts.

### Limitations of the approach and future research

This approach emphasises the need for diverse methodologies and data inputs in the context of urban planning to fully grasp the multifaceted nature of local urban climate and human thermal comfort. The research highlights the added value of interdisciplinary approaches in understanding and addressing the complex issue of thermal comfort, corroborating the results of previous studies such as Elnabawi and Hamza 2019 and Cureau et al. 2022. Using the low-cost, built-for-purpose, mobile thermal comfort mapping device, this study identified areas where subjective thermal votes collected using questionnaires were often closely correlated to the calculated UTCI based on the meteorological measurements. The combination of objective and subjective data provides a comprehensive view of thermal comfort, acknowledging that perceptions of comfort may not always align directly with quantitative measurements.

A key limitation of this study is the relatively small number of participants (*n* = 15), which limits the representativeness of the findings for the broader population of Dresden and the direct applicability of the method to other case study contexts. Additionally, only age ranges of the questionnaire participants were collected, rather than exact ages, which limits the ability to perform more detailed demographic analysis. Despite the small sample size, the high correlation between subjective votes and objective measurements demonstrates the utility of the low-cost device and its feasibility for identifying target areas for urban planning interventions in local studies. Future research with a larger participant pool and more detailed demographic data is recommended to improve the representativeness of the findings. Furthermore, questionnaires, short interviews, and other participatory methods, such as cognitive mapping, could also be employed in future studies to engage pedestrians and residents in assessing the selection of hot- and coldspots identified by the measurements, and to identify other areas of interest that may not be captured on the measurement route.

Another component that influences the usability and transferability of this type of linear measurement by a walking person is the prior selection of the measurement route as the influence of solar radiation on the calculated UTCI value depends largely on the shading of the walking route. In large sun-exposed squares, the differences in walking paths are negligible. However, in small structured areas with shade from trees and buildings, the measurements are only partially representative of the area. A sound knowledge of the area to be analysed, as well as the hypothesised hot and cold spots to be investigated, with a route selection based on this knowledge should form the basis for targeted measurement plan tailored to the problem at hand.

This paper presents a first step in which the basic usability of the low-cost device and low-resource methodology was tested for its ability to provide evidence-based urban planning insights. This approach should be further verified for applicability in other case study contexts. In the next step, the approach shall be discussed with stakeholders such as planning offices, to survey their needs in order to further refine the measurement approach for the creation of targeted evaluations, with accompanying citizen surveys to optimise the results for planning in practice.

## Conclusion

In this study, we presented the basis for an interdisciplinary, low-resource, and user-friendly thermal comfort mapping methodology designed to support evidence-based urban planning. The approach integrates objective meteorological data with subjective perceptions of thermal comfort, allowing for the identification of priority areas for intervention and suitable measures. By utilising the Universal Thermal Climate Index (UTCI), we identified priority areas requiring urban design and planning interventions to ameliorate as well as maintain thermal comfort.

The strengths of this methodology lie in its accessibility and practicality, enabling a broad spectrum of users - from urban planners to non-expert stakeholders - to assess thermal comfort in urban environments. The combination of objective meteorological data and subjective perceptions of thermal comfort enhances the depth of analysis, ensuring that interventions are not only scientifically grounded but also resonate with the lived experiences of urban residents.

However, the trade-off between scientific accuracy and usability means that while the approach is accessible, it may lack the precision of more complex climate modelling tools. Additionally, the reliance on UTCI, though effective for the context of this study, may not capture the full spectrum of thermal comfort factors in diverse urban settings.

Future work should aim to validate and refine this methodology by engaging with different stakeholders to gauge its reception and effectiveness in informing urban planning decisions. Furthermore, the study’s focus on a single urban context limits the generalisability of its findings, prompting the need for validation and refinement through broader application in diverse settings.

The need for a balance between scientific rigor and practical usability underscores the methodology’s potential as a tool for democratising urban climate studies. This study contributes significantly to the discourse advocating for evidence-based, participatory governance approaches, offering a pragmatic framework for addressing the challenges of urban heat in the pursuit of a more sustainable, liveable, and thermally comfortable urban future.

## Figures and Tables

**Table 2:**
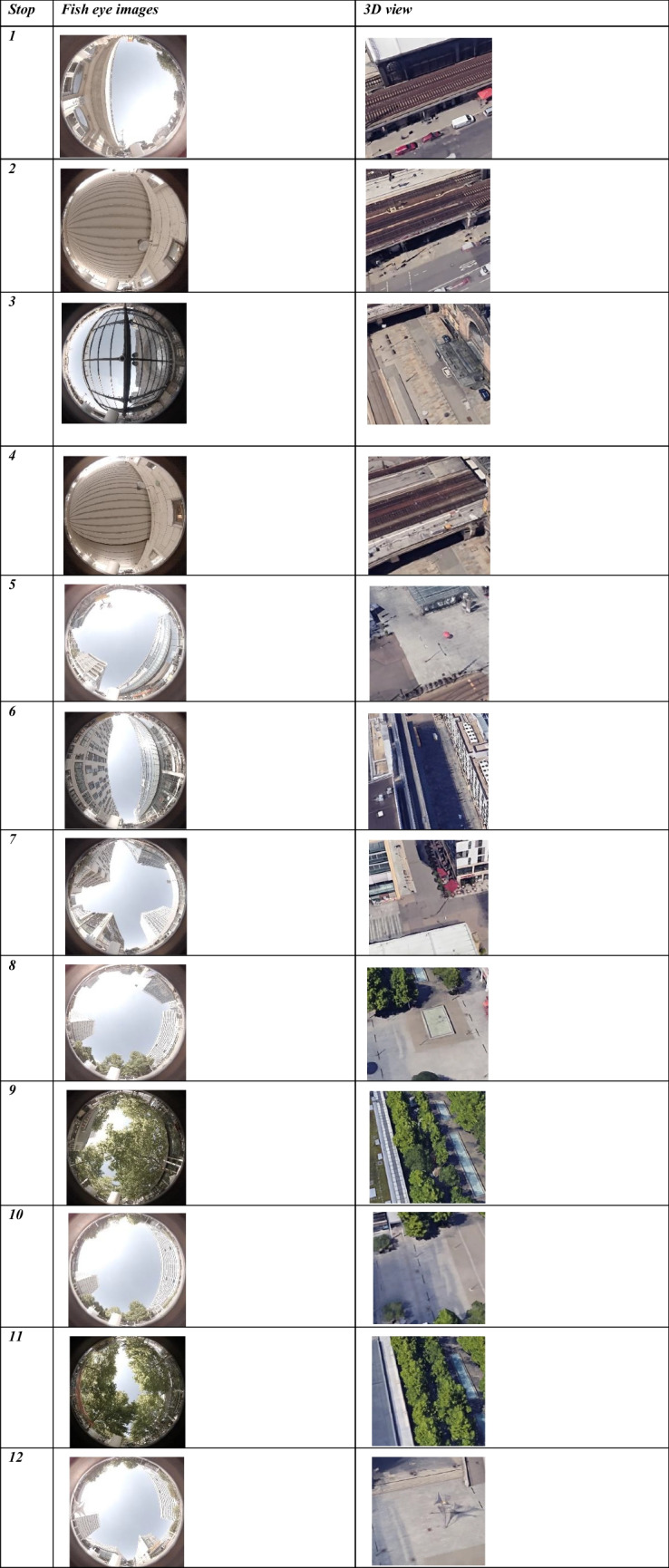
Fish eye images and 3D views of the 12 stops on the mobile questionnaire route (Fish eye image credit: Dr. Valeri Goldberg, TU Dresden. 3D image credit: Google, GeoBasis-DE/BKG (©2009))

**Table 3:**
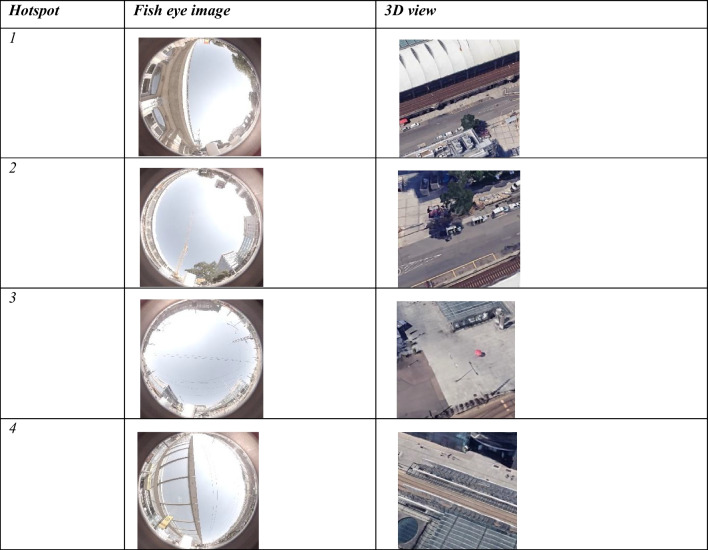
Fish eye images and 3D views of consistent hotspots (Fish eye image credit: Dr. Valeri Goldberg, TU Dresden. 3D image credit: Google, GeoBasis-DE/BKG (©2009))

**Table 4:**
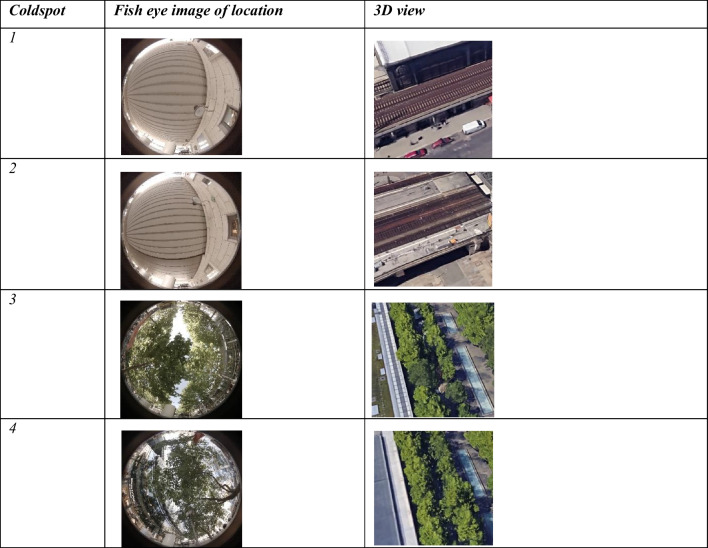
Fish eye images and 3D views of consistent coldspots (Fish eye image credit: Dr. Valeri Goldberg, TU Dresden. 3D image credit: Google, GeoBasis-DE/BKG (©2009))
