# Correction to: Pedestrian thermal comfort mapping for evidence-based urban planning; an interdisciplinary and user-friendly mobile approach for the case study of Dresden, Germany

**DOI:** 10.1007/s00484-025-02857-z

**Published:** 2025-01-28

**Authors:** Claire Gallacher, Denise Boehnke

**Affiliations:** 1 Leibniz Institute of Ecological Urban and Regional Development, Weberplatz 1, 01217 Dresden, Germany; 2 Faculty of Environmental Science, Dresden University of Technology, Dresden, Germany; 3 Division 4- Natural and Built Environment, Karlsruhe Institute of Technology (KIT), Karlsruhe, Germany

**Correction to: International Journal of Biometeorology**

10.1007/s00484-024-02830-2

The article was originally published without proper attribution for Figs. 1, 3, 5, and 6, which should have credited Google, Airbus, Maxar Technologies, and GeoBasis-DE/BKG. Additionally, Fig. 7, along with Tables 2, 3, and 4, was published without credit to Valeri Goldberg. The article has since been updated to include the appropriate credits: Figs. 1, 3, 5, and 6 are now credited to Google, Airbus, Maxar Technologies, and GeoBasis-DE/BKG, while Fig. 7 and Tables 2, 3, and 4 now appropriately credit Valeri Goldberg from TU Dresden. The original article has been corrected accordingly.

